# The use of Eculizumab and Tocilizumab in the treatment of Hyperhemolysis syndrome, a comprehensive literature review

**DOI:** 10.3389/fmed.2025.1651895

**Published:** 2025-09-16

**Authors:** Ali Aqel, Yousef Al-Asa’d, Salam Al-kindi, Jaafar Altouk, Abdullah Al Zayed, Abdulrahman Al-Abdulmalek, Mohamed A. Yassin

**Affiliations:** ^1^Hamad Medical Corporation, Internal Medicine, Doha, Qatar; ^2^Department of Haematology, Sultan Qaboos University, Muscat, Oman; ^3^Department of Internal Medicine, Bahrain Salmania Hospital, Manama, Bahrain; ^4^Qatif Central Hospital, Qatif, Saudi Arabia; ^5^Division of Hematology, McGill University, Montréal, QC, Canada; ^6^Department of Haematology, NCCCR, Hamad Medical Corporation, Doha, Qatar; ^7^College of Medicine, Qatar University, Doha, Qatar

**Keywords:** Eculizumab, HHS, Hyperhemolysis syndrome, monoclonal antibodies, Tocilizumab, transfusion reaction

## Abstract

Hyperhemolysis syndrome (HHS) is a rare but severe complication of red blood cell transfusion, characterized by the destruction of both the patient’s and donor’s red blood cells. This condition results in post-transfusion hemoglobin levels lower than pre-transfusion levels, often leading to profound anemia, tissue ischemia, and multiorgan failure. HHS predominantly affects individuals with hemoglobinopathies, particularly sickle cell disease. While the pathophysiology remains poorly understood, proposed mechanisms include bystander hemolysis via complement activation, suppression of erythropoiesis, macrophage-mediated RBC destruction. Refractory cases of HHS are managed with Eculizumab and Tocilizumab, targeting the complement pathway and macrophage activation, respectively. This review analyzed 22 reported cases of HHS identified through PubMed, Embase, and Google Scholar. Of these, 11 patients received Eculizumab, 10 received Tocilizumab, and 1 received both. The cohort had an mean age of 29.5 years, with 36.4% male and 63.6% female. Most patients had underlying hemoglobinopathies. Outcomes showed improvement in 18 patients without major side effects, while 1 patient showed no improvement, and 3 patients passed away. Despite promising results, concurrent use of other immune-modulating agents during treatment complicates attributing the observed efficacy to specific medications alone. Further studies are required to further evaluate the pathophysiology of HHS and assess the safety and effectiveness of these novel therapies.

## Introduction

Hyperhemolysis syndrome (HHS) is a rare but serious complication of red blood cell transfusion, characterized by the destruction of both the patient’s and donor’s RBCs. This typically results in a post-transfusion hemoglobin level lower than the pre-transfusion level (1). HHS is most observed in patients with hemoglobinopathies, particularly sickle cell disease. However, it has also been reported in other conditions, such as Human immunodeficiency virus (HIV) infection and lymphoma (1, 2). Although the exact pathophysiology of HHS remains unclear, several mechanisms have been proposed, including bystander hemolysis due to complement activation, suppression of erythropoiesis, and macrophage-mediated RBC destruction (3).

Hemolysis due to activation of the complement pathway, also known as bystander hemolysis, is considered one of the key mechanisms underlying HHS. In hyperhemolysis, IgG alloantibody binds donor RBCs and activates the complement system; activated complement components (notably C3/C5) then spill over locally and opsonize or lyse nearby autologous RBCs that were not the antibody’s target, hence the name bystander hemolysis. This mechanism is amplified in sickle cell disease and sickle RBCs appear unusually susceptible to complement-mediated damage (4). Eculizumab, a humanized monoclonal antibody, targets C5 to prevent its cleavage into C5a and the formation of the C5b-9 membrane attack complex, effectively inhibiting intravascular hemolysis (4). Due to this mechanism, Eculizumab has been used off-label in the management of HHS. While its FDA-approved indications include paroxysmal nocturnal hemoglobinuria and atypical hemolytic uremic syndrome (2, 5), ongoing research continues to explore its broader therapeutic potential. Clinical trials have assessed its efficacy in a range of conditions, including dense deposit disease, C3 nephropathy, solid organ transplant rejection, macular degeneration, neuromyelitis optica, myasthenia gravis, dermatomyositis, allergic asthma, antineutrophil cytoplasmic antibody vasculitis, and cold agglutinin disease (2).

Macrophage hyperactivation has also been implicated in the pathogenesis of HHS. Sickled RBCs exhibit increased expression of antigens and membrane phospholipids, like phosphatidylserine and surface-bound immunoglobulin G (IgG), enhancing recognition by hyperactivated macrophages and leading to extravascular hemolysis. Given the pro-inflammatory state in SCD, elevated cytokine levels may further stimulate macrophage-mediated destruction of both transfused and autologous RBCs, resembling mechanisms seen in cytokine release syndrome (CRS) and macrophage activation syndrome (MAS) (6, 7). Additionally, transfused RBCs exhibit reduced CD47 expression over time, increasing their susceptibility to macrophage erythrophagocytosis especially in the heightened inflammatory cytokines in patients with active sickle crises, as LEE et al. described that in their reported case all transfused RBC units were stored > 14 days (6). Tocilizumab is an IL-6 receptor antagonist which leads to a reduction in cytokine and acute phase reactant production and subsequently inhibiting macrophage activation. Due to probable similarities between HHS and CRS pathophysiology, it is being increasingly used in cases of refractory HHS with promising results. It has six FDA approved indications which are rheumatoid arthritis; giant cell arteritis; polyarticular juvenile idiopathic arthritis; systemic juvenile idiopathic arthritis; cytokine release syndrome associated with chimeric antigen receptor (CAR) T cell; and lastly COVID-19 as an emergency use authorization.

**Figure 1:**
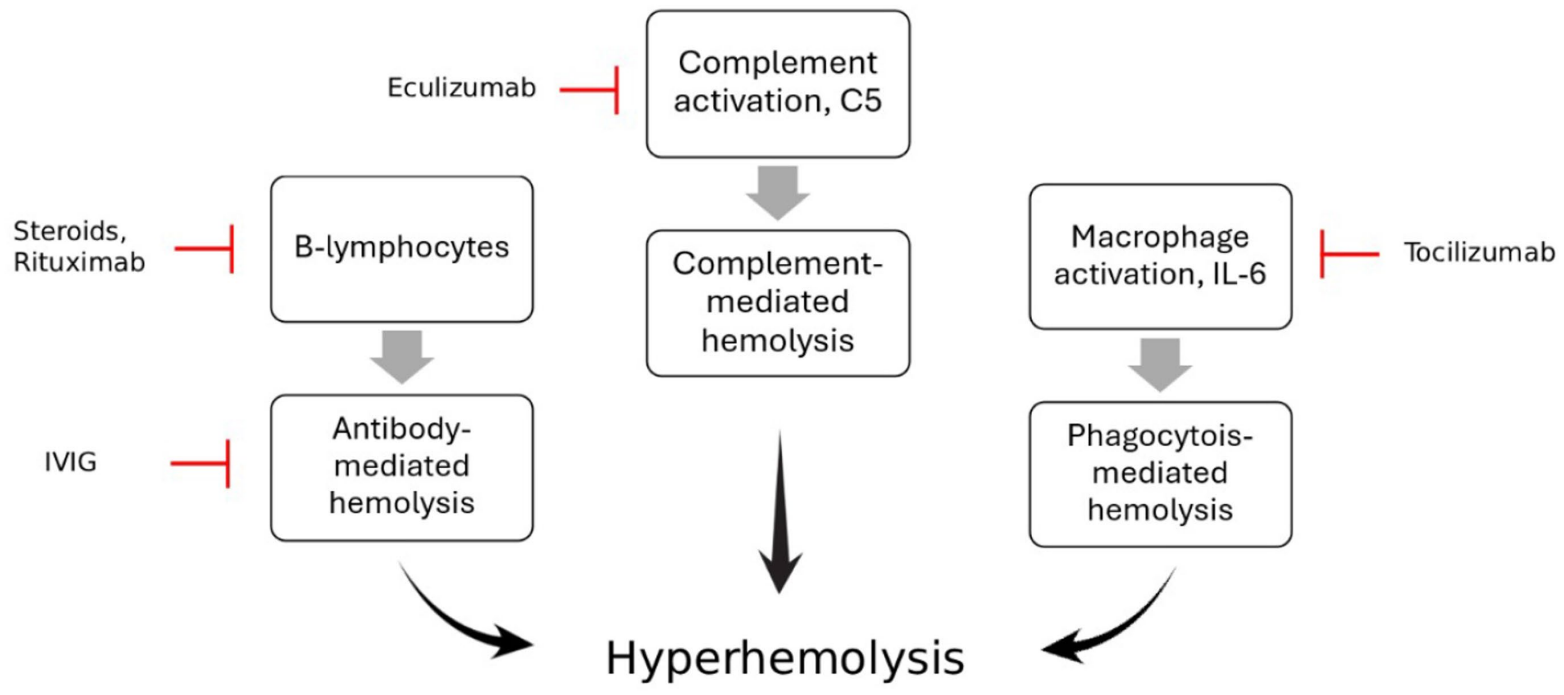
The pathophysiology of Hyperhemolysis syndrome and mechanism of action of different immune modulators such as Eculizumab, Tocilizumab and Rituximab, steroids and IVIG.

The rarity, severity, and knowledge gaps in Hyperhemolysis syndrome (HHS) with emerging therapeutic approaches, such as the off-label use of Eculizumab and Tocilizumab, make a literature review essential. This review brings together available information to assess current and new treatment options, and highlight the areas where more research is needed to help doctors manage this dangerous condition more effectively (Figure 1).

## Methodology

This literature review aims to examine the role of Eculizumab and Tocilizumab in the management of Hyperhemolysis syndrome (HHS) through a comprehensive literature review. The review was conducted using three electronic databases: PubMed, Embase and Google Scholar. The search focused on identifying relevant articles that describe the use of Eculizumab or Tocilizumab in managing HHS. Keywords and medical subject headings related to “Hyperhemolysis Syndrome, ““Hyperhemolysis,” “Eculizumab,” and “Tocilizumab” were used to find appropriate articles.

Studies were included based on specific criteria, including systematic reviews, meta-analyses, case series, and case reports that focused on patients diagnosed with HHS. Only studies that reported treatment with either Eculizumab or Tocilizumab were considered. Additionally, only full-text articles available in English were included in the review. Studies were excluded if they did not specifically address the use of these biologic agents in HHS management or lacked sufficient clinical details.

Relevant studies were screened based on titles and abstracts, followed by a full-text review to determine eligibility. Key clinical details, including patient demographics, treatment protocols, response to therapy, and outcomes, were extracted and synthesized. Since this is a qualitative review, no statistical analysis was performed.

As this study is based on publicly available literature and does not involve direct patient data collection, no ethical approval was required.

Basic statistical analysis, such as mean and standard deviation, were used to display some results in the results section. Analysis done using R software.

## Results

Upon reviewing the literature, 22 cases of HHS were identified in which patients treated with either Eculizumab or Tocilizumab, results are viewed in the following Tables 1–6.

**Table 1:** Basic characteristics and cause of admission of cases of Hyperhemolysis syndrome managed using either Eculizumab or Tocilizumab.

Case number	Age (Yrs)/sex	Indication for transfusion	Diagnosis/Hemoglobinopathy	History of transfusions before current presentation	H/o Alloantibodies	Reference
1	Teenager/F	vaso-occlusive crisis	sickle cell disease	N/A	N/A	Ita et al., 2024 (8)
2	9/M	worsening anemia	sickle cell disease (Hb SS)	N/A	No	Lapite et al., 2024 (9)
3	18/F	acute chest syndrome	sickle cell disease (Hb SS)	N/A	No	Lapite et al., 2024 (9)
4	30/F	worsening anemia	sickle cell disease	N/A	N/A	DALY et al., 2023 (10)
5	28/M	Covid pneumonia	sickle cell disease (Hb SS)	Yes	No	Green et al., 2023 (11)
6	39/F	worsening anemia	β-thalassemia intermediate	Yes	Yes	Cannas et al., 2021 (12)
7	24/M	vaso-occlusive crisis	sickle cell disease	N/A	N/A	Geiszler et al., 2021 (13)
8	28/F	vaso-occlusive crisis	sickle cell disease (Hb SS)	Yes	Yes	Unnikrishnan et al., 2019 (4)
9	21/F	vaso-occlussive crisis	sickle cell disease [Hb S (HBB: c.20A > T)/*β*-thalassemia (β-thal)]	N/A	N/A	Vlachaki et al., 2018 (5)
10	31/M	Pneumonia/Acute chest syndrome	SCD/thalassemia	Yes	Yes	ALKaabi et al., 2016 (14)
11	35/F	worsening anemia	sickle cell disease	Yes	Yes	Boonyasampant et al., 2015 (3)
12	58/F	worsening anemia	HIV and hepatitis C infections, COPD/ no Hemoglobinopathy	Yes	Yes	Gupta et al., 2014 (2)
13	30s/F	Suspected stroke	sickle cell disease (Hb SS)	Yes	No	Desai et al., 2023 (15)
14	21/M	vaso-occlusive crisis	sickle cell disease (Hb SS)	N/A	No	Desai et al., 2023 (15)
15	19/M	vaso-occlusive crisis	sickle cell disease (Hb SS)	Yes	Yes	Grant et al., 2023 (16)
16	29/F	worsening anemia	β-thalassemia	No	No	El Afifi et al., 2022 (17)
17	30s/M	Covid pneumonia, acute chest syndrome	sickle cell disease (Hb SS)	Yes	Yes	Fuja et al., 2022 (18)
18	33/F	elective surgery	sickle cell disease (Hb SS)	Yes	Yes	Meenan et al., 2022 (19)
19	21/F	worsening anemia	sickle cell disease (Hb SS)	Yes	No	Menakuru et al., 2022 (20)
20	47/F	Infected chronic right hip wound	Hemoglobin SC anemia	N/A	N/A	Rehman et al., 2021 (7)
21	36/F	worsening anemia	sickle cell disease	Yes	Yes	Lee et al., 2019 (6)
22	33/M	Acute chest syndrome	sickle cell disease (Hb SS)	Yes	No	Sivapalaratnam et al., 2019 (21)

H/O, History of. F, Female. M, Male. N/A, Not Applicable. HIV, Human immunodeficiency virus. Age not mentioned in the original report for case number 1.

**Table 2:** Presentation of Hyperhemolysis syndrome and details of transfusion.

Case number	HHS presentation (No. of days after transfusion)	Initial Hb level	Hb level after transfusion/ nadir	Number of transfusions recived (units)	DAT	New alloantibodies detected during HHS	Reference
1	3 days	6.2 gm/dL	2 gm/dL	12	Negative	No	Ita et al., 2024 (8)
2	6 days	5.3 gm/dL	4.2 gm/dL	1	Negative	No	Lapite et al., 2024 (9)
3	5 days	7.2 gm /dL	2.7 gm /dL	1	Negative	Yes	Lapite et al., 2024 (9)
4	4 days	7 gm/dL	2.3 gm/dL	1	N/A	N/A	DALY et al., 2023 (10)
5	1 day	5.8 gm/dL	1.7 gm/dL	3	N/A	N/A	Green et al., 2023 (11)
6	1 day	4.1gm/dL	2.7 gm/dL	2	Negative	no	Cannas et al., 2021 (12)
7	1 day	6.1 gm/dL	N/A	1	N/A	yes	Geiszler et al., 2021 (13)
8	6 days	5.8 gm/dL	1.8 gm/dL	2	Positive	Yes	Unnikrishnan et al., 2019 (4)
9	2 days	6.9 gm/dL	5.4 gm/dL	2	Negative	No	Vlachaki et al., 2018 (5)
10	4 days	7.3 gm/dL	6.2 gm/dL	1	N/A	N/A	ALKaabi et al., 2016 (14)
11	14 days	7.4gm/L	3.6 gm/dL	2	positive	Yes	Boonyasampant et al., 2015 (3)
12	3 days	Hct of 17.9%	Drop of Hct to below 10%	4	Negative	Yes	Gupta et al., 2014 (2)
13	6 days	8.0 gm/dL	2.9 gm/dL	3	Positive	No	Desai et al., 2023 (15)
14	5 days	7 gm/dL	3.3 gm/dL	3	Positive	No	Desai et al., 2023 (15)
15	Not mentioned	8.8 gm/dL	3.4 gm/dL	Not mentioned (multiple)	Positive	N/A	Grant et al., 2023 (16)
16	12 days	7.8 gm/dL	4.8 gm/dL	3	Negative	No	El Afifi et al., 2022 (17)
17	14 days	8.4 gm/dL	4.4 gm/dL	5	Positive	Yes	Fuja et al., 2022 (18)
18	11 days	10.1 gm/dL	4.6 gm/dL	N/A	Weak positive	Yes	Meenan et al., 2022 (19)
19	1 day	5.2 gm/dL	4.5 gm/dL	2	Negative	N/A	Menakuru et al., 2022 (20)
20	9 days	9.4 gm/dL	2.5 gm/dL	5	N/A	N/A	Rehman et al., 2021 (7)
21	8 days	5.5 gm/dL	2.1 gm/dL	Not mentioned (multiple)	Negative	No	Lee et al., 2019 (6)
22	8 days	6.3 gm/dL	3.2 gm/dL	12 units exchange transfusion	Negative	No	Sivapalaratnam et al., 2019 (21)

HHS, Hyperhemolysis Syndrome. No., Number. Hb, Hemoglobin. HcT, Hematocrit. DAT, Direct Antiglobulin Test. N/A, Not Applicable. Mmol/L Millimole per Liter. gm/dL, gram per deciliter. gm/L, gram per liter.

**Table 3:** Laboratory findings during hemolysis Hyperhemolysis syndrome cases.

Case number	LDH	Bilirubin	Retic count	haptoglobin	Presence of splenomegaly	Reference
1	Increased	Increased	Decreased	Decreased	Not mentioned	Ita et al., 2024 (8)
2	Increased	Increased	Decreased	Decreased	Not mentioned	Lapite et al., 2024 (9)
3	N/A	N/A	Decreased	N/A	Not mentioned	Lapite et al., 2024 (9)
4	Increased	Increased	Increased	Decreased	Not mentioned	DALY et al., 2023 (10)
5	Increased	Increased	Decreased	N/A	Not mentioned	Green et al., 2023 (11)
6	Increased	Increased	Decreased	Decreased	Not mentioned	Cannas et al., 2021 (12)
7	Increased	Increased	N/A	N/A	Not mentioned	Geiszler et al., 2021 (13)
8	Increased	Increased	Decreased	N/A	Not mentioned	Unnikrishnan et al., 2019 (4)
9	Increased	Increased	Decreased	N/A	Not mentioned	Vlachaki et al., 2018 (5)
10	Increased	Increased	Decreased	Decreased	Not mentioned	ALKaabi et al., 2016 (14)
11	Increased	Increased	Decreased	Decreased	Not mentioned	Boonyasampant et al., 2015 (3)
12	Increased	N/A	N/A	Decreased	Yes	Gupta et al., 2014 (2)
13	N/A	N/A	N/A	N/A	Not mentioned	Desai et al., 2023 (15)
14	N/A	N/A	Decreased	N/A	Not mentioned	Desai et al., 2023 (15)
15	Increased	Increased	N/A	N/A	Not mentioned	Grant et al., 2023 (16)
16	Increased	Increased	Decreased	Decreased	Not mentioned	El Afifi et al., 2022 (17)
17	Increased	Increased	increased	Decreased	Not mentioned	Fuja et al., 2022 (18)
18	Increased	Increased	Decreased	N/A	Not mentioned	Meenan et al., 2022 (19)
19	Increased	Increased	Decreased	N/A	Not mentioned	Menakuru et al., 2022 (20)
20	Increased	N/A	N/A	N/A	Yes	Rehman et al., 2021 (7)
21	Increased	Increased	Decreased	Decreased	Not mentioned	Lee et al., 2019 (6)
22	Increased	Increased	N/A	N/A	Not mentioned	Sivapalaratnam et al., 2019 (21)

LDH, Lactate Dehydrogenase. N/A, Not Applicable.

**Table 4:** Use of steroids and IVIG in cases of Hyperhemolysis syndrome managed using either Eculizumab or Tocilizumab.

Case number	Steroid use	IVIG	Reference
1	Methylprednisolone given (dosing not mentioned)	Given (dosing not mentioned)	Ita et al., 2024 (8)
2	Methylprednisolone 30 mg/kg daily for 3 days	1 g/kg in 2 doses	Lapite et al., 2024 (9)
3	Methylprednisolone 1 g IV tapered on 10 days	1 g/Kg daily for 4 days	Lapite et al., 2024 (9)
4	not given	Given (dosing not mentioned)	DALY et al., 2023 (10)
5	Methylprednisolone 1 g IV (duration not mentioned)	1 g/kg	Green et al., 2023 (11)
6	Methylprednisolone 250 mg IV daily (duration not mentioned)	2 g/kg/day for 5 days in 2 intervals, one before Eculizumab and one after Eculizumab	Cannas et al., 2021 (12)
7	Methylprednisolone given (dosing not mentioned)	Given (dosing not mentioned)	Geiszler et al., 2021 (13)
8	Methylprednisolone 125 mg IV daily tapered through hospital course	1 g/kg once	Unnikrishnan et al., 2019 (4)
9	Methylprednisolone 40 mg/day (duration not mentioned)	1 g/kg per day for 2 days	Vlachaki et al., 2018 (5)
10	Dexamethasone (dosing not mentioned)	single dose (dosing not mentioned)	ALKaabi et al., 2016 (14)
11	Not given	Not given	Boonyasampant et al., 2015 (3)
12	Methylprednisolone 125 mg IV (duration not mentioned)	Not given	Gupta et al., 2014 (2)
13	Methylprednisolone 0.5 g IV (duration not mentioned)	1 g/kg for 2 days	Desai et al., 2023 (15)
14	Methylprednisolone 0.5 g IV for 2 days	1 g/kg for 2 days	Desai et al., 2023 (15)
15	Methylprednisolone (dosing not mentioned)	Given (dosing not mentioned)	Grant et al., 2023 (16)
16	Methylprednisolone 1 g IV followed by Prednisone 1 mg/kg tapering	0.4 g/kg for 5 days	El Afifi et al., 2022 (17)
17	Not given	Not given	Fuja et al., 2022 (18)
18	Methylprednisolone 0.5 g IV for 2 days	0.4 g/kg for 5 days	Meenan et al., 2022 (19)
19	prednisone 4 mg/kg for 4 days	0.5 g/kg for 4 days	Menakuru et al., 2022 (20)
20	Methylprednisolone (dosing not mentioned)	Given (dosing not mentioned)	Rehman et al., 2021 (7)
21	Methylprednisolone 1 g IV for 5 days	0.4 g/kg for 4 days	Lee et al., 2019 (6)
22	Methylprednisolone 0.5 g IV for 3 days	0.4 g/kg for 5 days	Sivapalaratnam et al., 2019 (21)

IVIG, Intravenous immunoglobulin. mg/Kg, milligram per kilogram. g/Kg, gram per kilogram. g, gram. g/kg, Gram per kilogram. mg, milligrams.

**Table 5:** Monoclonal antibody used and other treatments in cases of Hyperhemolysis syndrome.

Case number	Monoclonal antibody used	Other treatments/interventions	Reference
1	600 mg of Eculizumab IV once	N/A	Ita et al., 2024 (8)
2	600 mg of Eculizumab IV once	Folic acid, Vitamin b12, EPO	Lapite et al., 2024 (9)
3	900 mg of IV Eculizumab twice (on Day of Admission 4 and 10)	EPO, folic acid, 750 mg/kg on Day of Admission 1 and 14	Lapite et al., 2024 (9)
4	Eculizumab given (dose not mentioned)	N/A	DALY et al., 2023 (10)
5	900 mg of IV Eculizumab once	EPO	Green et al., 2023 (11)
6	900 mg of IV Eculizumab twice at a 7-day interval	Rituximab (375 mg/m2 on days 1, 4, 8, and 12) (before Eculizumab), EPO, IV Iron	Cannas et al., 2021 (12)
7	2 doses of Tocilizumab and 2 doses of Eculizumab	N/A	Geiszler et al., 2021 (13)
8	900 mg of IV Eculizumab once	HBOC-201 (Hemopure), EPO, IV iron, and vitamin B12	Unnikrishnan et al., 2019 (4)
9	900 mg of IV Eculizumab once	Rituximab IV 500 mg/once (before Eculizumab)	Vlachaki et al., 2018 (5)
10	900 mg of IV Eculizumab twice at a 7-day interval	EPO, Rituximab given after Eculizumab	ALKaabi et al., 2016 (14)
11	1,200 mg of Eculizumab weekly for 4 weeks starting from day 3 of admission followed by every 2 weeks maintenance starting on Treatment Day 29	rituximab, 375 mg/m2, was given weekly for 4 weeks starting on Treatment Day 3, after the initiation of Eculizumab	Boonyasampant et al., 2015 (3)
12	600 mg of Eculizumab IV once	EPO	Gupta et al., 2014 (2)
13	Tocilizumab 8 mg/kg for 4 days	Rituximab 375 mg/m2, Eculizumab 900 mg	Desai et al., 2023 (15)
14	Tocilizumab 8 mg/kg for 4 days	N/A	Desai et al., 2023 (15)
15	Tocilizumab (dosing not mentioned)	Plasma exchange	Grant et al., 2023 (16)
16	Tocilizumab 8 mg/kg for 2 days	Darbepoetin alfa, oral iron, vitamin B12, folic acid, rituximab 375 mg/m2	El Afifi et al., 2022 (17)
17	Tocilizumab 8 mg/kg for 1 day	N/A	Fuja et al., 2022 (18)
18	Tocilizumab 8 mg/kg for 4 days	EPO	Meenan et al., 2022 (19)
19	Tocilizumab (dosing not mentioned)	4,000 IU of EPO, IV folate, IV iron, IV vitamin B12	Menakuru et al., 2022 (20)
20	Tocilizumab (dosing not mentioned)	Darbepoetin, splenic embolization, rituximab, plasmapheresis	Rehman et al., 2021 (7)
21	Tocilizumab 8 mg/kg for 4 days	EPO, cyanocobalamin, folic acid, and as last resort hemoglobin-based oxygen carrier-201 (HBOC-201)	Lee et al., 2019 (6)
22	Tocilizumab 8 mg/kg for 2 days	N/A	Sivapalaratnam et al., 2019 (21)

N/A: Not Applicable. EPO: Erythropoietin. IV, Intravenous. mg/Kg, milligram per kilogram. mg, milligrams. mg/m2, milligram per square meter. HBOC, Hemoglobin-based oxygen carriers. IU, International unit.

**Table 6:** Outcome of cases of Hyperhemolysis syndrome managed using either Eculizumab or Tocilizumab.

Case number	Length of hospital stay (days)	Outcome	Reference
1	N/A	Improvement	Ita et al., 2024 (8)
2	9	Improvement after 1 day of Eculizumab dose	Lapite et al., 2024 (9)
3	17	Improvement after 1 day of Eculizumab dose	Lapite et al., 2024 (9)
4	N/A	Improvement	DALY et al., 2023 (10)
5	9	passed away	Green et al., 2023 (11)
6	77	Improvement from Day 7 of Eculizumab Injection	Cannas et al., 2021 (12)
7	19	Improvement	Geiszler et al., 2021 (13)
8	49	Improvement	Unnikrishnan et al., 2019 (4)
9	N/A	Improvement after 3 days of Eculizumab injection	Vlachaki et al., 2018 (5)
10	9	Improvement	ALKaabi et al., 2016 (14)
11	N/A	Improvement from day 7 of Eculizumab injection	Boonyasampant et al., 2015 (3)
12	33	Failure of Eculizumab to prevent intravascular hemolysis after transfusion	Gupta et al., 2014 (2)
13	18	Improvement	Desai et al., 2023 (15)
14	35	Improvement	Desai et al., 2023 (15)
15	N/A	Passed away	Grant et al., 2023 (16)
16	62	Improvement	El Afifi et al., 2022 (17)
17	27	Improvement (Hb reached 9.1)	Fuja et al., 2022 (18)
18	17	Improvement	Meenan et al., 2022 (19)
19	14	Improvement (Hb reached 8.3)	Menakuru et al., 2022 (20)
20	12	Passed away	Rehman et al., 2021 (7)
21	23	Improvement (Hb reached 8.8)	Lee et al., 2019 (6)
22	13	Improvement	Sivapalaratnam et al., 2019 (21)

N/A, Not Applicable. Hb, Hemoglobin.

The mean age of the patients was 29.5 years (Standard deviation (SD) ± 11.1 years), with 36.4% male and 63.6% female. All patients, except for Case 12, had underlying hemoglobinopathies. In case 12 HHS occurred in the context of HIV and hepatitis C infection. As previously described in the literature, the most common underlying hemoglobinopathy was sickle cell disease compromising 81.8% of the cases in this review, including two patients with concurrent thalassemia (Table 1).

Most patients were admitted to the hospital and received transfusion either for worsening anemia or vaso-occlusive crisis (81.8%), The mean number of transfusion units was 3.4 units (SD ± 3.27), with multiple patients only receiving one unit. On average, HHS developed 5.9 days post-transfusion, with a range of 1 to 14 days (Table 2).

Reticulocytopenia and elevated hemolysis markers were present in all cases except for Case 17, highlighting the critical role of reticulocyte percentage in differentiating HHS from delayed transfusion reactions (Table 3).

All patients received standard therapy of methylprednisolone and Intravenous Immunoglobulin (IVIG) for a range of 1 to 5 days, except for case number 11 that received rituximab and Eculizumab without steroids or IVIG (Table 4).

Among the 22 patients, 11 patients received Eculizumab (50%), 10 patients received Tocilizumab (45.5%) and 1 patient received both Eculizumab and Tocilizumab (4.5%). Dosing duration varied significantly between all cases, emphasizing the need of more studies that look into dosing of these novel therapies in HHS. For example, six patients received only one dose of Eculizumab and one case received one dose Tocilizumab with most of them improving, while other cases received up to 4 doses of either Eculizumab or Tocilizumab, and in case number 7 the patient received 2 doses of Tocilizumab and 2 doses of Eculizumab (Table 5). Among these patients, six patients also received rituximab either before or after Eculizumab or Tocilizumab, two patients received plasmapheresis, and two patients received hemoglobin-based oxygen carrier-201 (HBOC-201) (Table 6).

As seen in Table 6, there were 18 out of the 22 cases that improved, hemolysis subsided and hemoglobin levels improved after treatment, on the other hand, one patient did not improve, and three patients passed away. In case number five, the patient passed away after he developed cardiac arrest secondary to severe hypotension from femoral hematoma at the site of the apheresis catheter, while the other two patients developed refractory hyperhaemolysis with significant drop of hemoglobin leading to multiorgan failure and death (7, 16).

## Discussion

HHS typically manifests within seven to fourteen days post-transfusion, presenting with pain, fever, jaundice, and hemoglobinuria (7). However, in most of the cases that we reported HHS occurred within 1 week after transfusion. In patients with sickle cell disease, HHS is associated with fever, vaso-occlusive crises, severe anemia, and laboratory evidence of hemolysis, including hemoglobinuria, hyperbilirubinemia, and elevated lactate dehydrogenase (LDH). Reticulocytopenia and hyperferritinemia are additional hallmark features. Further transfusions can exacerbate hemolysis, whereas recovery is indicated by an increase in hemoglobin and reticulocyte count, accompanied by normalization of ferritin levels (20).

HHS is classified into acute and delayed forms. The acute variant occurs within 7 days of transfusion, while the delayed form develops beyond this period. These forms can be distinguished through direct antiglobulin testing (DAT) and alloantibody screening. Acute HHS is characterized by a negative DAT and the absence of alloantibodies, suggesting a mechanism driven by macrophage activation. Recent studies indicate that in acute, antibody-negative HHS, RBC destruction is primarily mediated by activated macrophages, with both extravascular and intravascular hemolysis contributing to disease pathogenesis (6). In contrast, delayed HHS is associated with a positive DAT and alloantibody presence, implicating antigen–antibody interactions in the initial destruction of transfused RBCs. Subsequently, cytokine release (e.g., IL-1 and IL-6) recruits macrophages, which further destroy autologous RBCs via adhesion-mediated mechanisms (20). This classification is not well defined in the literature, and among the cases we reported, few cases had positive DAT despite occurring within 7 days after transfusion, which contradicts putting a boundary line between acute and delayed cases. Notably, only 35.2% of HHS episodes are linked to newly formed alloantibodies or autoantibodies (18).

Hyperhemolysis syndrome (HHS) is a severe condition that often leads to profound anemia, which can result in tissue ischemia and multiorgan failure. In addition to anemia-related complications, direct heme-induced organ injury and cytokine-mediated inflammation further contribute to the poor prognosis of this disease (4). For instance, Green et al. (11) reported a case of HHS in a patient with sickle cell disease and a concurrent COVID infection. Despite multiple treatment attempts, including steroids, IVIG, Eculizumab, and EPO, the patient succumbed to the illness (11). Similarly, Rehman et al. (7) described a case of recurrent HHS in which the patient did not survive despite receiving IVIG, methylprednisolone, Tocilizumab, darbepoetin, splenic embolization, and plasmapheresis. These cases highlight the life-threatening nature of HHS and the challenges in its management. In certain high-risk situations, such as pregnancy, hyperhemolysis poses an even greater threat, endangering both the mother and the fetus (12). Cannas et al. (12) reported a case of a pregnant woman with *β*-thalassemia intermedia who developed HHS. She underwent treatment with steroids, IVIG, rituximab, and Eculizumab, ultimately discharged after a prolonged 77-day hospitalization (12).

The mainstay of HHS treatment includes avoidance of further blood transfusions, corticosteroids and intravenous immunoglobulin (IVIG), the latter two suppress macrophage activation and mitigate hemolysis (7, 20). Avoiding further RBC transfusions is essential, as additional transfusions can paradoxically worsen anemia through continued RBC destruction. Most cases of HHS demonstrate stabilization of hemoglobin within five days of initiating therapy; however, refractory cases may result in hypoxia-induced multiorgan failure and death in the absence of salvage interventions (6). Erythropoietin has been employed in HHS management to stimulate erythropoiesis, with suggested dosing ranging from 250 to 800 units/kg/dose thrice weekly or 40,000–60,000 units of recombinant human erythropoietin weekly (17, 20). In refractory cases, alternative treatments are being increasingly utilized. Rituximab, a CD20-targeting monoclonal antibody; Eculizumab, a complement inhibitor; and Tocilizumab, a macrophage inhibitor, have demonstrated efficacy in promoting recovery. The variability in treatment response highlights the complexity of HHS, emphasizing the need for a tailored, multi-faceted management approach.

Eculizumab effectively inhibits terminal complement activation by targeting the C5 component, preventing its cleavage into C5a and the formation of the C5b-9 membrane attack complex. This mechanism not only halts intravascular hemolysis but also suppresses C5a, a potent inflammatory mediator, making it a valuable treatment option for cold antibody-mediated hemolysis (2, 3). Additionally, its ability to block complement-mediated destruction of precursor RBCs is evident through the observed increase in reticulocyte counts following treatment initiation (3). Beyond its role in hemolysis, Eculizumab has demonstrated safety and efficacy during pregnancy, leading to favorable maternal and fetal outcomes. A study by Kelly et al. (22) reported that pregnant patients with Paroxysmal Nocturnal Hemoglobinuria who received Eculizumab had high fetal survival rates and a low incidence of maternal complications (22). Similarly, Cannas et al. (12) described a case of a pregnant woman with *β*-thalassemia intermedia who developed HHS. Despite fetal prematurity, she responded well to Eculizumab and achieved a successful recovery with a good fetal outcome (12). Eculizumab has also proven to be an effective treatment for HHS in the pediatric population. Lapite et al. (9) published a case series of two sickle cell patients with HHS, in which hemolysis was halted within just 1 day of Eculizumab administration (9). These findings highlight the drug’s rapid and profound impact, reinforcing its role as a critical therapeutic option for managing complement-mediated hemolysis across different patient populations.

Eculizumab has not consistently demonstrated success in halting intravascular hemolysis. Gupta et al. (2) reported a case of HHS without hemoglobinopathy where a single 600 mg intravenous dose failed to stop hemolysis possibly due to insufficient dosing. Similarly, Green et al. (11) documented a case in which a patient with sickle cell disease, concomitant HHS, and COVID-19 infection succumbed despite receiving a 900 mg intravenous dose. Notably, most cases in the table received IVIG and steroids before Eculizumab, with some also undergoing Rituximab treatment. This raises the question of whether hemolysis cessation was solely due to Eculizumab or the combined effect of multiple immune modulators.

Tocilizumab, an IL-6 receptor antagonist, has shown promise in HHS treatment by mitigating macrophage activation. It is well-tolerated and has demonstrated efficacy in MAS, CRS, and severe COVID-19 pneumonia. Multiple case reports indicate successful resolution of HHS with Tocilizumab administration without reported complications. Given its relatively lower cost and wider clinical experience, particularly in resource-limited settings, Tocilizumab may serve as an alternative to Eculizumab. The optimal dosing regimen remains uncertain, though a CRS-based strategy involving four doses at least 8 h apart has been employe. Treatment usually shows rapid ferritin response that can guide further administration and possibly dosing. In most cases that used Tocilizumab, there was an observed rise in IL-6 levels following Tocilizumab therapy, this may reflect effective blockade of IL-6R, leading to increased circulating unbound cytokine (6, 7, 15, 18, 20). Off note, L-6 is the principal cytokine driving C-reactive protein (CRP) production, and Tocilizumab administration is associated with reduced CRP levels. Therefore, clinical caution is warranted when interpreting CRP values in patients receiving Tocilizumab (15). Combination therapy with rituximab and Tocilizumab has been evaluated in other autoimmune conditions, such as autoimmune encephalitis, demonstrating favorable efficacy and safety profiles (23).

Plasma-to-RBC exchange transfusion with concurrent standard care may be considered in recurrent HHS cases (7).

Further studies are required to determine optimal treatment strategies and establish standardized therapeutic protocols for managing HHS effectively. Moreover, other novel treatments can be considered in future research and trials such as pegcetacoplan, a C3 inhibitor of the alternative complement pathway, that was used and showed a promising result in autoimmune hemolytic anemia (24).

## Limitations

While the reported cases provide valuable insights into the use of Tocilizumab and Eculizumab in refractory cases of HHS, several limitations must be acknowledged. These include the rarity of the disease itself which was reflected in the small number of cases that received Tocilizumab or Eculizumab, this poses a question regarding generalizability of the treatment results. A small number of cases also makes it difficult to conduct randomized controlled trials to compare these novel therapies with the standard treatment. Moreover, the concurrent administration of other immune-modulating agents during treatment complicates the ability to pinpoint the precise medication responsible for halting hemolysis.

## Conclusion

HHS is a rare but potentially life-threatening condition. While standard therapy is often effective in managing the syndrome, there remain challenging cases that are refractory to conventional treatments. Emerging evidence from available case reports highlights promising outcomes with the use of Tocilizumab and Eculizumab in such refractory cases. In addition, these agents offer an opportunity to further understand the underlying pathophysiology of HHS. However, the rarity of the disease poses significant challenges to research. Future studies are essential to deepen our understanding of its pathophysiology and to evaluate the effectiveness and safety profiles of these novel medications.
